# Potential for Cell-Mediated Immune Responses in Mouse Models of Pelizaeus-Merzbacher Disease

**DOI:** 10.3390/brainsci3041417

**Published:** 2013-09-30

**Authors:** Cherie M. Southwood, Bozena Fykkolodziej, Fabien Dachet, Alexander Gow

**Affiliations:** 1 Center for Molecular Medicine and Genetics, Wayne State University School of Medicine, Detroit, MI 48201, USA; E-Mails: csouthwo@med.wayne.edu (C.M.S.); bfykkol@med.wayne.edu (B.F.); fdachet@med.wayne.edu (F.D.); 2 Carman and Ann Adams Department of Pediatrics, Wayne State University School of Medicine, Detroit, MI 48201, USA; 3 Department of Neurology, Wayne State University School of Medicine, Detroit, MI 48201, USA

**Keywords:** cytokines, chemokines, myeloid, lymphoid, microarrays, *rumpshaker*, *myelin synthesis-deficient*

## Abstract

Although activation of the innate and adaptive arms of the immune system are undoubtedly involved in the pathophysiology of neurodegenerative diseases, it is unclear whether immune system activation is a primary or secondary event. Increasingly, published studies link primary metabolic stress to secondary inflammatory responses inside and outside of the nervous system. In this study, we show that the metabolic stress pathway known as the unfolded protein response (UPR) leads to secondary activation of the immune system. First, we observe innate immune system activation in autopsy specimens from Pelizaeus-Merzbacher disease (PMD) patients and mouse models stemming from *PLP1* gene mutations. Second, missense mutations in mildly- and severely-affected *Plp1*-mutant mice exhibit immune-associated expression profiles with greater disease severity causing an increasingly proinflammatory environment. Third, and unexpectedly, we find little evidence for dysregulated expression of major antioxidant pathways, suggesting that the unfolded protein and oxidative stress responses are separable. Together, these data show that UPR activation can precede innate and/or adaptive immune system activation and that neuroinflammation can be titrated by metabolic stress in oligodendrocytes. Whether or not such activation leads to autoimmune disease in humans is unclear, but the case report of steroid-mitigated symptoms in a PMD patient initially diagnosed with multiple sclerosis lends support.

## 1. Introduction

The immune system is an important player in the pathophysiology of a number of neurodegenerative diseases [1,2]. Neuroinflammation includes activation of central nervous system (CNS)-resident microglia or infiltrating blood-derived macrophages and secretion of a variety of cytokines and chemokines into the parenchyma [3]. A wide variety of insults including infection, trauma, ischemia and metabolic stress, are known to be associated with neuroinflammatory processes and there is increasing evidence that microglia/macrophages and several key cytokines play both pathologic and neuroprotective roles in pathogenesis [4,5,6].

The unfolded protein response (UPR) is also an important cell stress response that likely modulates the pathology in degenerative CNS disorders. This response stems from membrane-bound kinase- and transcription factor-based sensors in the endoplasmic reticulum that detect changes in protein and lipid fluxes through the secretory pathway. These sensors signal to the nucleus to induce molecular chaperone expression, activate transcription factor cascades and transiently suppress global protein translation to maintain cell homeostasis [7].

A crucial feature of UPR activation involves the dependence on misfolded protein loading in the secretory pathway; the greater the rate of accumulation of aberrantly-folded proteins, the greater is the activation of the UPR and the more severe is the pathology [8,9]. This loading principle also has been demonstrated *in vivo*, where reducing the accumulation of mutant *Plp1* gene products in the secretory pathway of myelinating oligodendrocytes from *rsh* mice mitigates their phenotype from severe ataxia, seizures and death by four weeks of age, to survival beyond eight weeks [10].

Activation of the UPR has been observed in human post-mortem samples of patients suffering from a number of diseases, which suggests an important role for this signaling pathway as a cell response mechanism during neurodegeneration [11,12,13]. The etiologies of these diseases may involve protein aggregation or disrupted protein trafficking associated with the expression of coding region mutations in familial forms or noxious environmental stimuli, and have been reported to activate the UPR in all major CNS cell types [14,15,16,17,18,19].

For example, Pelizaeus-Merzbacher disease (PMD) is an *X*-linked recessive neurodegenerative leukodystrophy largely caused by three genetic lesions in the PROTEOLIPID PROTEIN 1 (*PLP1*) gene: deletions, duplications and missense/nonsense mutations. UPR activation underlies pathogenesis in humans and mice with *PLP1* coding region mutations [8,9,19,20]. *PLP1* gene deletion and duplication causes disease through poorly understood mechanisms not involving UPR activation at early stages in the disease process [21]. However, duplications in mice are associated with late stage induction of an inflammatory response [22] which may subsequently activate the UPR in oligodendrocytes via proinflammatory cytokine signaling [23].

In previous studies, we demonstrated that coding region mutations in the *PLP1* gene cause the encoded proteins, PLP1 and DM-20, to misfold and accumulate in the endoplasmic reticulum (ER) of transfected cells and oligodendrocytes leading to activation of the UPR and an increased incidence of apoptosis [8,19,20,24]. Furthermore, we established a prognostic test in transfected cells [9] that can be used to predict disease severity for PMD patients and animal models of PMD from ER accumulation of both PLP1 and DM-20 (severe disease), or PLP1 but not DM-20 (mild disease). However, our studies have not revealed major differences in UPR activation or changes in gene expression between severe and mild disease [19]. This leaves open the question of why disease severity for all missense mutations is not relatively constant, and has prompted us to explore additional disease mechanisms that are secondary to metabolic stress in oligodendrocytes and may modify disease progression, such as immune cell activation and inflammation.

Increasingly, inflammatory and UPR signaling pathways appear to be interconnected [25]. The UPR induces inflammatory signaling, such as the NFκB or jun-kinase (JNK) pathways [26,27,28,29] and, conversely, inflammatory cytokines such as IL-1, IL-6, TNF-α and IFN-γ activate UPR signaling [30]. Potentially, induction of either pathway may generate a positive-feedback loop that exacerbates disease. For example, IFN-γ [23,31] activates the UPR during experimental allergic encephalomyelitis (EAE) in mice, which is an *in vivo* model of select clinical features in multiple sclerosis (MS). However, few studies have focused on interactions between the UPR and inflammatory responses in these disorders.

Herein, we demonstrate activation of the innate immune system in PMD autopsy specimens from *PLP1* missense and duplication patients as well as in two missense mutant mouse models of PMD, *rsh* and *msd* mice, and *Plp1*-overexpressor mice. In addition, we observe distinct immune-associated expression profiles in optic nerve microarray data from the missense mutant mice, which may be associated with protective or pathogenic environments for oligodendrocytes or other cell types and modulate the disease course and severity. These profiles suggest that greater disease severity is associated with a proinflammatory environment in *msd* but not *rsh* mice, which has been previously suggested for *Plp1* overexpressor mice [22,32]. Together, these data demonstrate a nonimmune-mediated mechanism of disease in the CNS, whereby genetic lesions in a myelin-specific structural protein activate the UPR and trigger an innate immune response that, in severe cases, could broaden to include inflammatory or autoimmune responses mediated by the adaptive immune system. These data are most significant in the context of diseases such as MS because they raise the possibility of an etiology by which neuroinflammation may evolve secondarily to an underlying metabolic pathophysiology.

## 2. Results and Discussion

Because of the functional cross-talk between the UPR and immune system that has been revealed in recent years, and the observation that immune signaling can activate the UPR in oligodendrocytes [33], it seems reasonable to postulate that activation of the UPR may also lead to modulation of immune signaling in the local environment. Major cell types that perform local surveillance functions and are mediators of immune signaling in the CNS include microglia/macrophages and astrocytes. These cell types are rapidly activated by diverse forms of CNS pathology and, in response, secrete cytokines and chemokines into the parenchyma. Astrocytes are widely known to be activated *in vivo* by the pathophysiological processes stemming from mutant *Plp1* expression in animals and PMD patients, but microglia/macrophages have been studied in less detail.

### 2.1. Microglia/Macrophage Activation Is Variable in Pelizaeus-Merzbacher Disease Patients

**Figure 1:**
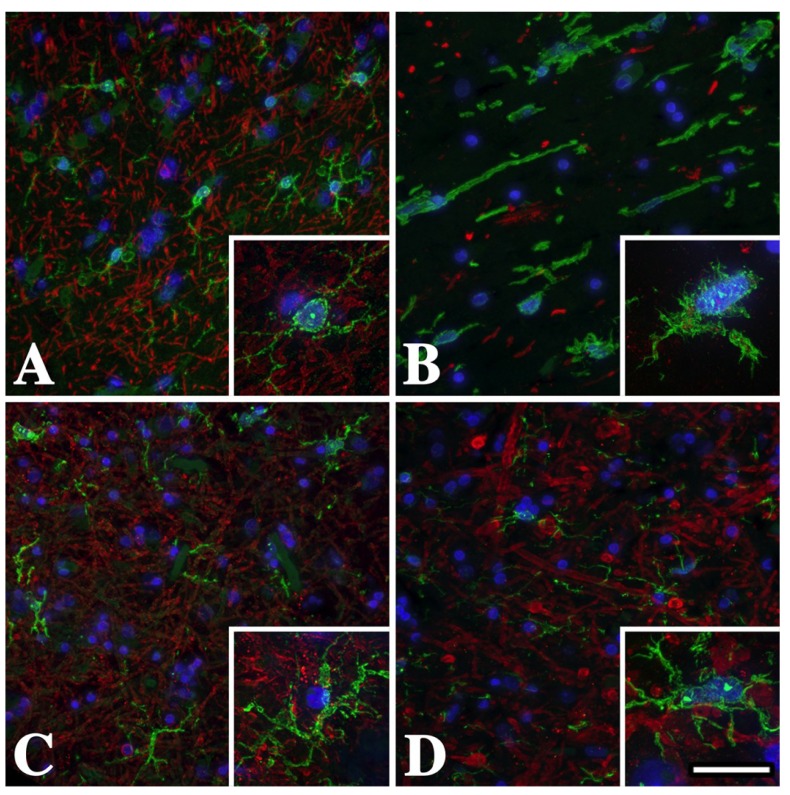
Temporal lobe subcortical white matter microglia/macrophages in male Pelizaeus-Merzbacher disease (PMD) patients are activated and express Iba-1. (**A**) Iba-1^+^ (green) resting microglial morphology in the U-fibers from a normal individual who suffered a myocardial infarct. Microglial processes are long, thin and branching (inset). There are abundant myelin basic protein (MBP^+^) myelinated axons (red) in this region. (**B**) Activated microglia/macrophages with short thickened processes and amoeboid morphology in the U-fibers from a severely affected PMD patient with a T42I missense mutation in PROTEOLIPID PROTEIN 1 (PLP1). The lack of MBP staining in this section reflects the virtual absence of white matter in this patient. (**C**) Microglia/macrophage morphology in the U-fibers from this mildly-affected *PLP1*-null patient is more similar to resting (**A**) than to activated (**B**) cells, although there is some thickening of the processes (inset). (**D**) Microglia/macrophage morphology in the U-fibers from this moderately affected *PLP1* gene duplication patient is intermediate between the missense mutation (**B**) and the *PLP1*-null patient (**C**). Scale bar: 50 μm, insets 20 μm.

To explore our hypothesis that metabolic stress in oligodendrocytes can activate microglia/macrophages, we examined autopsy tissue sections of cortex of the U-fibers in the temporal lobes of four patients: a 75 year old female control who had suffered a myocardial infarct unrelated to neurological disease; a male PMD patient with severe disease arising from the *PLP1* gene missense mutation, T42I; a male PMD patient with mild disease caused by a null allele of the *PLP1* gene; and a severely affected *PLP1* gene duplication male patient (Figure 1). There are few reliable antibodies that specifically label activated human microglia/macrophages from formalin-fixed tissues and we have relied on morphology to determine if these cells are resting or activated. This approach is common in published studies [34].

Cortical sections from patients were labeled with antibodies against myelin basic protein (MBP) to reveal white matter tracts and the calcium-binding protein, Iba-1, which is expressed strongly in resting and activated microglia/macrophages. Immunofluorescence staining of U-fibers from the normal control is shown in Figure 1A. Myelin sheath staining is robust in this region and resident microglia are numerous and well defined. The detailed morphology of these cells is apparent in the inset and shows long fine branching processes (ramified morphology) radiating from the cell body with stereotypic features of a resting (non-activated) cell [35]. In contrast, microglia/macrophages from a PMD patient with severe disease (Figure 1B) show typical features of activation, with short thick non-branching processes and an overall amoeboid appearance. The virtual absence of MBP labeling (red) in this field, which is largely reduced to debris, reflects extensive white matter pathology and the virtual absence of myelin.

Microglia/macrophage morphology in the mildly affected *PLP1-*null PMD patient (Figure 1C) appears intermediate between the resting cells of the control (Figure 1A) and the severe patient (Figure 1B). Thus, although the cell body in the Figure 1C inset remains small, the cell processes are thicker. Microglia/macrophages from a *PLP1* duplication patient with moderate disease (Figure 1D) are also intermediate between the control and missense patient and similar to Figure 1C. Together, these data suggest that primary oligodendrocyte pathology stemming from distinct *PLP1* gene mutations has differential effects on microglia/macrophage morphology and potentially may activate these cells in unique ways or to variable extents.

### 2.2. Microglia/Macrophage Activation Varies with Disease Severity in *Plp1* Mutant Mice

**Figure 2:**
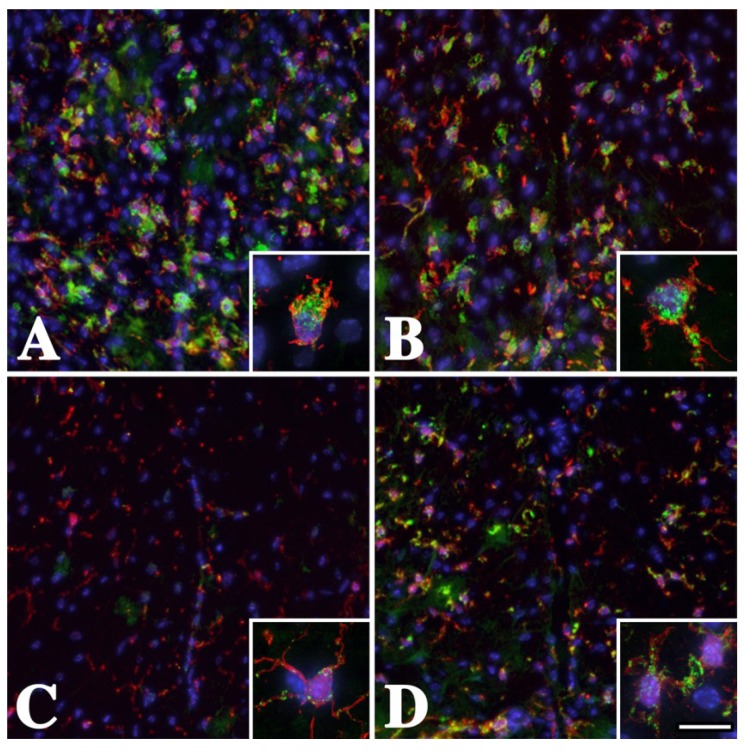
White matter microglia/macrophages are activated in the dorsal funiculi of male *Plp1* mutant mice. Cervical spinal cord sections from (**A**) mildly-affected P16 *rsh* (**B**) severely-affected P16 *msd* (**C**) subclinical P25 *Plp1*#66 heterozygous (**D**) moderately-affected P25 *Plp1*#66 homozygous mice labeled with antibodies against Iba-1 (red) and CD68 (green). Many Iba-1 positive microglia/macrophages with swollen cell bodies and short processes are labeled with anti-CD68 antibodies in *rsh* and *msd* mice, which is indicative of activated cells. The insets in these panels show the canonical activated morphology revealed with anti-Iba-1 antibodies and strong CD68 staining. In contrast, there is little evidence for broad CD68 labeling in *Plp1*#66 heterozygous mice in panel C, which is consistent with previous data that immune cells are rarely activated [22]. Activation of microglia/macrophages is apparent for *Plp1*#66 homozygous mice in panel D, which exhibit an intermediate level of CD68 labeling. Scale bar: 95 μm, insets 15 μm.

To determine if the morphological changes observed in microglia/macrophages from PMD tissue are recapitulated in mouse models of this disease, which are amenable to detailed experimental analysis, we immunolabeled transverse cervical spinal cord sections from two missense *Plp1* mutant mice, *rsh* (mild disease) and *msd* (severe disease), as well as heterozygous (sub-clinical disease) and homozygous (moderate disease) *Plp1*#66 overexpressor mice with anti-Iba-1 antibodies (red). Microglia/macrophage changes in the dorsal funiculi of *rsh, msd* and homozygous *Plp1*#66 mice are similar and show markedly shortened process morphology (Figure 2A,B,D).

Skoff and colleagues [22] have previously demonstrated microglia/macrophage activation in adult homozygous *Plp1#66* mice and our data are consistent with their findings. In heterozygous *Plp1*#66 mice, microglia/macrophages are similar to wild type (not shown) with small cell bodies, although the processes show some thickening and are not extensively ramified. These data suggest that the overall response of human and mouse microglia/macrophages to *PLP1* gene mutations is conserved and that further analyses in mice may be useful for inferring pathophysiological processes in PMD patients.

In addition to Iba-1 staining, we labeled mouse spinal cord sections with antibodies against CD68 (Figure 2) to determine if morphological changes in microglia/macrophages are accompanied by the activation of these cells [36,37,38]. CD68^+^ cells (green) are observed in all four genotypes, which indicates that changes in microglia/macrophage morphology and activation are commensurate processes in *Plp1* mutant mice.

Quantification of the proportions of Iba-1^+^ white matter microglia/macrophages that are also CD68^+^ are shown in Table 1. These data were generated from pooled cell counts in the dorsal column, ventral column and lateral funiculus of multiple spinal cord sections from each mouse. There are no significant regional differences in the proportions of CD68^+^ microglia/macrophages within each spinal cord region or genotype and CD68 labeling appears to be stronger in *rsh* tissue than in *msd*, *Plp1*#66 homozygotes or wild type. Roughly 20% of Iba-1^+^ cells in wild type mice weakly express this marker (not shown), while almost all microglia/macrophages in the mutants are CD68^+^ and are likely activated. Neither this antibody (ED-1) nor a second monoclonal anti-CD68 antibody (clone #514H12, AbD Serotec, Oxford, UK) label microglia/macrophages in human sections, so we cannot confirm activation of these cell types in PMD patients; however, the archetypal morphological changes [34] that we observe in Figure 1 are consistent with the mouse data and with an activated state.

**Table 1:** Proportion of cervical spinal cord Iba-1^+^ microglia/macrophages in dorsal column, ventral column and lateral funiculus that are activated (CD68^+^).

Genotype	Proportion of CD68^+^/Iba-1^+^ cells (%) ^a^	# Mice
Wild type	22 ± 1.9	3
*rsh*	90 ± 1.0	3
*msd*	70 ± 4.1	3
*Plp1*#66 ^b^	82	1

^a^ 400–500 Iba^+^ cells counted per mouse; ^b^ homozygous for the *Plp1*#66 transgene.

**Figure 3:**
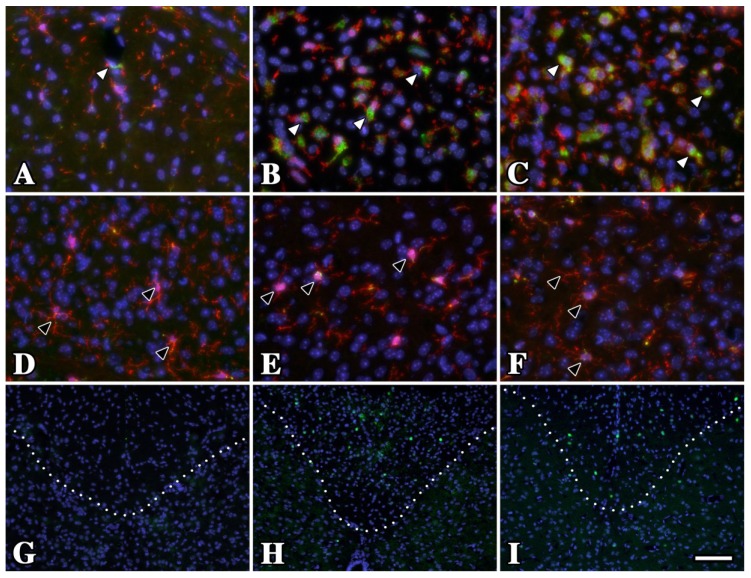
Oligodendrocytes in gray matter from P16 *rsh* and *msd* mice do not undergo a UPR and microglia/macrophages exhibit resting morphology. (**A**–**C**) Iba1 (red) and CD68 (green) antibody labeling in dorsal spinal cord white matter tracts of P16 wild type (**A**) *rsh* (**B**) and *msd* (**C**) mice. Although the morphology and CD68 staining is characteristic of resting microglia/macrophages in wild type mice (white arrowheads), these cells are activated in the *Plp1* mutants. (**D**–**F**) In contrast to white matter regions, microglia/macrophages have a resting state phenotype in the adjacent substantia gelatinosa (gray matter) from wild type and mutant mice (black arrowheads). (**G**–**I**) A major difference between these regions in *rsh* and *msd* mice is the relative abundance of oligodendrocytes undergoing an unfolded protein response (UPR), which is evident by the large number of CHOP^+^ cells (green) in white matter (above the dotted line) compared to gray matter (below). We have previously shown that 100% of CHOP^+^ cells in these mutants are oligodendrocytes [19]. Dotted lines mark the white/gray matter boundary. Scale bar in I: 100 μm.

In contrast to the major white matter tracts from brain (Figure 2) and cervical spinal cord (Figure 3B,C) from *rsh* and *msd* mice, in which microglia/macrophages are robustly activated, these cells are predominantly non-activated in adjacent gray matter of the substantia gelatinosa (Figure 3E,F), and appear similar to the ramified morphology and low CD68 expression of wild type cells (Figure 3A,D).

These data are significant for two reasons. First, they indicate that microglia/macrophage activation in *Plp1* mutant mice is driven by local interstitial factors, for example oligodendrocytes undergoing the UPR or apoptosis [19,20], which would be expected if these immune cells limit their surveillance and reaction to stimuli within discrete domains of the parenchyma. Second, although oligodendrocytes are distributed throughout gray matter and myelinate the low density of larger caliber axons within, they infrequently induce the UPR or undergo apoptosis in *Plp1* mutants (Figure 3E,F). As a consequence, the local factors in white matter to which microglia/macrophages respond are more likely to be UPR activation and death of myelinating oligodendrocytes than synthesis of mutant PLP1 per se, which occurs in both white and gray matter regions.

### 2.3. Optic Nerve Microarray Analysis in *rsh* and *msd* Mice Reveal Similarities in Expression Profiles

**Figure 4:**
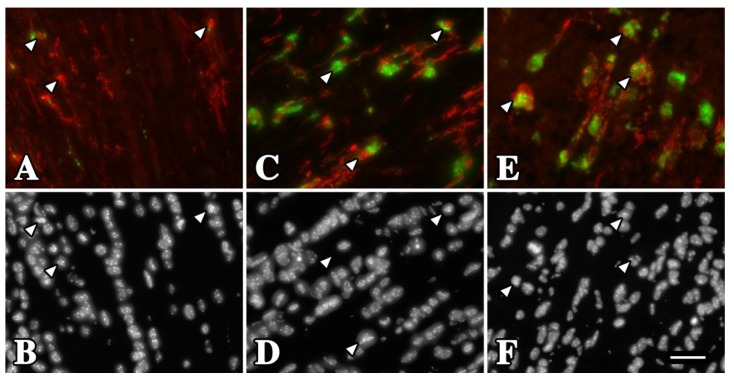
Activation of *rsh* and *msd* microglia/macrophages in optic nerve at P16. (**A**) Longitudinal section from wild type mouse reveals Iba-1^+^ microglia/macrophages (red) with non-activated morphology (arrowheads). Most of these cells do not express CD68 or express the protein at low levels (green). (**B**) DAPI staining showing the nuclei of the microglia/macrophages in (**A**). (**C**, **E**) Iba-1^+^ microglia/macrophages from *rsh* (**C**) and *msd* (**E**) mice exhibit an activated morphology with enlarged cell bodies and thickened processes. Most of these cells express CD68 at high levels, which is localized to perinuclear regions. (**D**, **F**) DAPI staining of the fields in (**C**, **E**). Scale bar in F: 30 μm.

We observe morphological variability in activated microglia/macrophages between the PMD patients (Figure 1) and between the mouse samples (Figure 2) such that the more extreme morphological changes are associated with severe symptoms. Accordingly, we hypothesized that gene expression profiles might also vary according to symptoms and, we performed a whole mouse genome microarray analysis using optic nerves from *rsh* and *msd* mice at P16. This tissue was chosen for three reasons: it is a relatively late myelinating tract that is heavily damaged by the effects of mutant PLP1 expression; oligodendrocyte density is high and accounts for approximately 50% of total cells, and; neuronal cell bodies are remotely located in the retina. The time point of P16 was chosen because the disease is well developed in the animals at this age but has not reached the final degenerative phase that decimates the proliferative population of oligodendrocyte progenitors in *msd* mice [20]. Similar to spinal cord and other myelinated tracts in brain (not shown), microglia are activated in optic nerve from *rsh* and *msd* mice as revealed by their morphology and CD68 expression (Figure 4). Together with Figure 1, Figure 2 and Figure 3, these data demonstrate that immune cell activation is widespread in white matter tracts throughout the CNS.

Initially, we compared the microarray expression profiles of wild type littermates from each of the *rsh* and *msd* colonies. We anticipated these profiles would be indistinguishable because both colonies have been maintained on the same B6C3 F1 hybrid background for more than 10 years (see Methods). Indeed, less than 0.01% of the microarray probes are differentially expressed (defined as greater than 1.5-fold change, *FDR* < 0.1) for wild type mice in the *rsh* versus *msd* colonies, which provides a considerable measure of confidence in the overall quality of sample collection, RNA purification and microarray analysis. Moreover, the mouse strain is known to influence the phenotype of *rsh* mice [39]; thus, our data demonstrates that we can exclude genetic background as a confounding factor in comparisons between *rsh* and *msd* mice.

**Figure 5:**
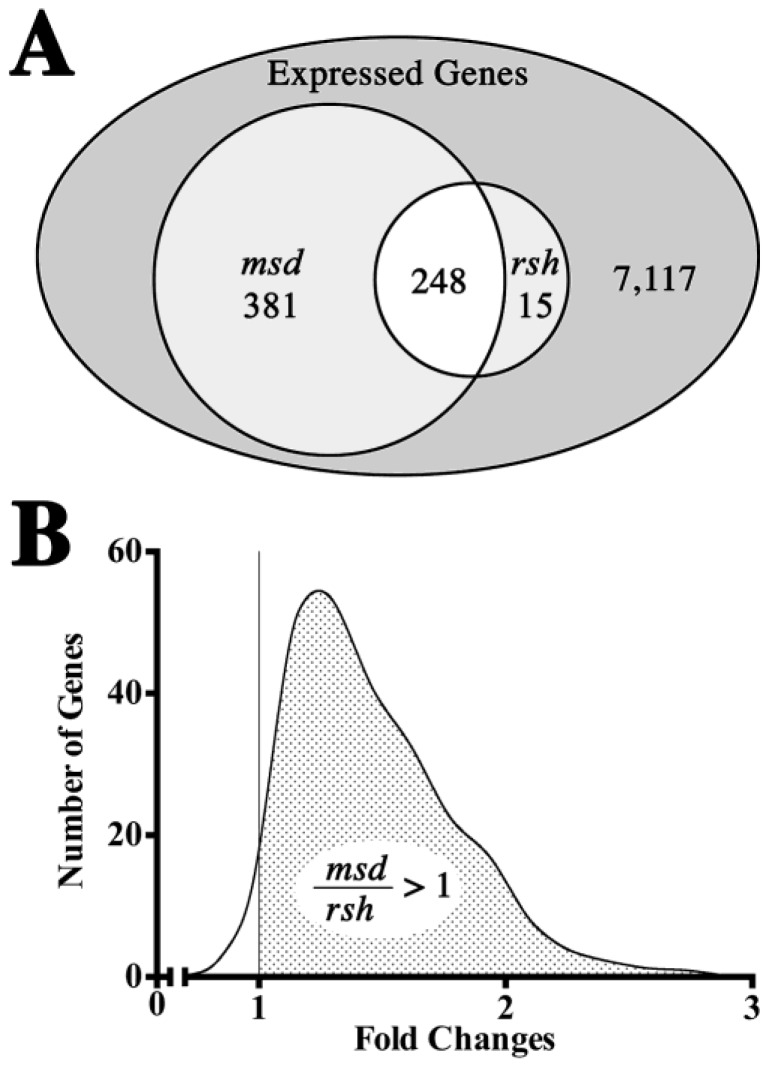
General characteristics of P16 optic nerve expression microarrays from *rsh* and *msd* mice. (**A**) Venn diagram for 7761 non-redundant expressed genes out of a total of 41,165 probes represented on the Agilent microarrays. Of 644 genes with dysregulated expression (>1.5-fold expression over control mice, *FDR* < 0.1) in the mutant mice, 248 genes are common to both *rsh* and *m*sd, 15 are unique to *rsh* and 381 genes are unique to *msd*. (**B**) Continuous distribution of fold changes (up and down) for 248 genes that are dysregulated in both *rsh* and *msd*. The asymmetry of this distribution about the unitary fold change, *X* = 1 (vertical line) shows that most genes are dysregulated to a greater extent in *msd* than in *rsh* (*FDR* < 0.1). Within the stippled region, 85.6% of genes are changed at least 10% more (*FDR* < 0.1) in *msd* than *in rsh.*

A Venn diagram of expression changes for *rsh* and *msd* mice compared to their respective wild type littermates is shown in Figure 5A. Of the 41,165 probes represented on the microarrays, 7761 genes are expressed at least two-fold above median local background levels and 644 of these genes (8.3% of expressed genes, *FDR* < 0.1) are dysregulated by at least 1.5-fold (up or down) in the mutant mice. This vastly exceeds the differences between the wild type colonies and we conclude that gene expression profiles between *rsh* and *msd* mice reflect disease-dependent changes rather than genetic drift in the background strains.

An important feature to emerge in this analysis from the perspective of pathogenesis is the similarity of the expression profile changes in *rsh* mice compared to *msd*. While 38.5% of the dysregulated genes are common to *rsh* and *msd*, 59.2% of the dysregulated genes are unique to *msd* but only 2.3% of the genes are unique to *rsh*. Cast in a different light, 94.3% of genes dysregulated in *rsh* are also dysregulated in *msd* which indicates that the *rsh* gene set is almost entirely a subset of *msd*. Such concordance suggests that disease severity rather than a distinct disease mechanism is the major factor contributing to gene expression changes, even though *msd* mice suffer from recurrent severe seizures beginning around P21 and live only 3–4 weeks while *rsh* mice have only mild seizures between 1 and 3 months of age and live a normal lifespan.

A comparison of expression fold changes for the genes dysregulated in *rsh* and *msd* mice is shown in Figure 5B. Fold change ratios greater than unity reflect expression of those genes that are more strongly affected by the *msd* phenotype than the *rsh* phenotype. The pronounced asymmetry of this graph about the unitary fold change, *X* = 1 (*i.e*., gene expression changed to the same extent in *rsh* and *msd*), indicates that the vast majority of genes dysregulated in both mutants (98.3%) change to a greater extent in *msd*. Taken together with the similarity of the *rsh* and *msd* profiles, these data demonstrate at the level of transcription that *rsh* mice approximate a milder form of the *msd* phenotype, at least at P16.

### 2.4. Validation of Microarray Gene Expression Data for UPR and Myelin Genes

We and others have determined previously that myelin genes are down regulated and UPR pathway genes are induced in *rsh* and *msd* mice compared to controls [19,20,24,40,41]. To validate gene expression data from the optic nerve microarrays, we compared fold changes (against controls) in these mutants for 10 genes with corresponding Taqman PCR and northern blotting data (Table 2). Expression fold-changes for the genes on the microarrays were statistically significant (*FDR* < 0.1) and one sample t-tests of expression fold changes from Taqman PCR/northern blotting data confirmed that all of these genes were statistically different in the mutant mice compared to controls (*p* < 0.05). In several instances (e.g., for *Atf3* and *Trib3*), the absolute values of the microarray fold changes differ from the Taqman results. Such inconsistencies are not unexpected, and are often observed between qPCR and microarrays, most likely because signal compression artifacts in the microarray output and because the locations of microarray and Taqman probes in different exons or 3' untranslated regions along the mRNA can influence assay sensitivity.

**Table 2:** Expression of UPR genes in optic nerve microarrays from *rsh* and *msd* mice compared to previous northern blotting data in spinal cord.

Function	Gene	Expression Fold-Change Compared to Controls				
		Microarrays ( *FDR* < 0.1) P16 Optic Nerve		Taqman PCR/Northern Blot P16 Spinal Cord		
		*rsh*	*msd*		*rsh*	*msd*
Myelin	*Plp1*	0.44 ± 0.08	0.23 ± 0.05		0.31 ± 0.03^ a^	0.08 ± 0.01 ^a^
	*Mbp*	0.56 ± 0.08	0.39 ± 0.03		0.42 ± 0.05^ a^	0.17 ± 0.01 ^a^
	*Mog*	0.59 ± 0.07	0.37 ± 0.03		0.35 ± 0.02^ a^	0.08 ± 0.01 ^a^
	*Cnp1*	0.67 ± 0.12	0.46 ± 0.04		*n.m.*	*n.m.*
	*Cldn11*	0.66 ± 0.13	0.35 ± 0.09		*n.m.*	*n.m.*
	*Mag*	0.63 ± 0.10	0.41 ± 0.03		*n.m.*	*n.m.*
UPR	*Chop*	2.1 ± 0.40	1.9 ± 0.40		4.2 ± 1.0 ^a^	3.5 ± 1.0 ^a^
	*Atf3*	1.5 ± 0.20	1.7 ± 0.30		13.8 ± 3.8 ^a^	7.5 ± 1.5 ^a^
	*Atf4*	1.3 ± 0.20	1.3 ± 0.10		1.4 ± 0.07 ^a^	1.5 ± 0.02 ^a^
	*Noxa*	*n.d.*	*n.d.*		1.79 ± 0.18 ^a^	1.67 ± 0.41 ^a^
	*Trib3*	3.2 ± 0.40	3.0 ± 0.70		72 ± 19 ^a^	58 ± 10 ^a^
	*4eBP1*	1.8 ± 0.01	1.8 ± 0.01		2.8 ± 0.4 ^a^	4.0 ± 0.5 ^a^
	*BiP*	1.7 ± 0.37	1.7 ± 0.35		1.7 ± 0.4 ^a^	1.7 ± 0.2 ^a^
	*Calreticulin*	1.3 ± 0.36	1.4 ± 0.46		*n.m.*	1.2 ^b^

^a^ derived from Taqman PCR; ^b^ derived from northern blotting; *n.m.*, not measured; *n.d.*, not detected.

### 2.5. Optic Nerve Microarray Data Reveal Disease-Severity Dependent Interferon-Responsive Gene Induction in *rsh* and *msd* Mice

**Figure 6:**
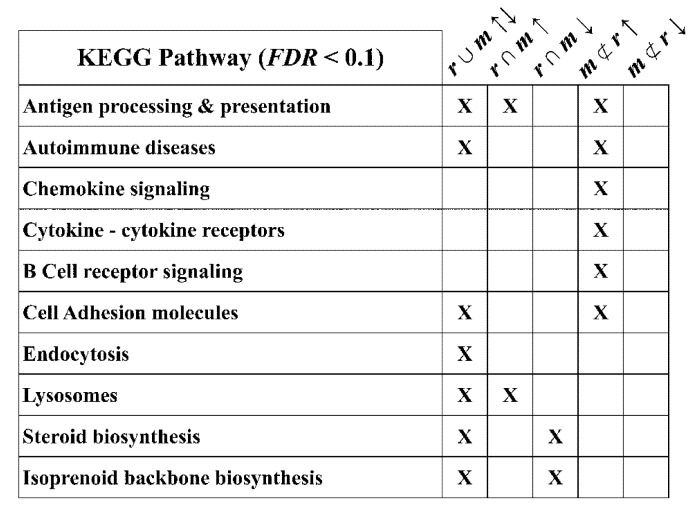
KEGG pathways enriched in dysregulated genes from *rsh* and *msd* mice. A DAVID bioinformatics analysis of dysregulated subsets of genes from *rsh* and *msd* microarrays identifies a number of KEGG pathways that are statistically enriched in *rsh* and/or *msd* mice (“X”; *FDR* < 0.1). The subsets of dysregulated genes examined are: all *rsh* and *msd* changed up and down (*r* ∪ *m ↑↓*), common to *rsh* and *msd* changed up or down (*r* ∩ *m ↑*; *r* ∩ *m ↓*) and unique to *msd* changed up or down (*m* ⊄ *r ↑*; *m* ⊄ *r ↓*). The Endosome and Lysosomes pathways were not evaluated in the Results section because of their generality to all cell types.

To further examine the optic nerve microarray data for aspects of known biology that we observe using other techniques (e.g., Figure 2), we used DAVID functional gene enrichment [42] to identify KEGG pathways that include genes differentially expressed in the mutant mice with respect to wild type littermates. We examined five expression categories as input gene subsets to DAVID: all *rsh* and *msd* changed up and down (*r* ∪ *m ↑↓*), common to *rsh* and *msd* changed up or down (*r* ∩ *m ↑*; *r* ∩ *m ↓*) and unique to *msd* changed up or down (*m* ⊄ *r ↑*; *m* ⊄ *r ↓*). The major output KEGG pathways identified are summarized in Figure 6.

From the perspective of the microglial activation that we observe in tissue sections from PMD patients and *Plp1* mutant mice (Figure 1 and Figure 2), the identification of KEGG immune system-related pathways from the DAVID ontology analysis is satisfying. Three of these pathways are retrieved using the list of all changed genes from *rsh* and *msd* mice but they are most robustly enriched using the induced gene subsets (Figure 6). Further characterization of the induced genes using a crude cluster analysis as a function of chromosomal location highlights a 2 Mb hotspot on chromosome 17 (chr17:34, 066, 052-36, 299, 338, mm9), within which 20% of the expressed genes represented on the microarrays are induced in *rsh* and/or *msd* mice (Table 3). Furthermore, most of these genes are induced to a greater extent in *msd* than in *rsh* (*FDR* < 0.1). This hotspot is located in the major histocompatibility complex (MHC) class I region encompassing the interferon-induced *H2-K*, *-L*, *-Q* and *-T* loci [43] and the peptide cotransporter, *Tapbp1*, which is necessary for loading peptides into the endoplasmic reticulum for binding to MHC molecules. The *Aif1* gene, which encodes the Iba 1 protein induced in microglia from *rsh* and *msd* mice (Figure 2), is also located in this genomic region.

Several important genes from the nearby MHC class II region on chromosome 17 are also induced in the mutants. These include the *Psmb8* and *Psmb9* (as well as *Psmb10* and *Psme2* on chromosomes 14 and 8) genes, which are interferon-inducible subunits of the immunoproteasome and are necessary to process peptides prior to loading into the endoplasmic reticulum [44]. Thus, induction of the major interferon-inducible molecular components necessary to generate functional MHC trimolecular complexes and present cell surface antigenic peptides to the adaptive immune system indicates broad immune pathway activation in the mutants. Further, in light of the expression level data for these genes we find that the magnitude of activation of the antigen presentation machinery is directly proportional to disease severity.

**Table 3:** Expression fold changes of chromosome 17 MHC I and II localized immune-related genes in microarray data for *rsh* and *msd* mice.

Gene	Fold Change		Gene	Fold Change	
	*rsh*	*msd* ^a^		*rsh*	*msd* ^a^
*H2-Ab1*	1.21 ± 0.22 ^c^	1.53 ± 0.17 ^b^	*H2-T22*	1.52 ± 0.16 ^a^	2.21 ± 0.56 ^b^
*H2-K1*	1.67 ± 0.39 ^a^	2.69 ± 0.49 ^b^	*H2-T23*	1.6 ± 0.31 ^a^	2.85 ± 0.27 ^b^
*H2-K2*	1.37 ± 0.13 ^c^	2.17 ± 0.25 ^b^	*Tapbp1*	1.27 ± 0.15 ^c^	1.62 ± 0.18 ^b^
*H2-L*	1.37 ± 0.26 ^c^	2.22 ± 0.15 ^b^	*Aif1*	2.09 ± 0.55 ^a^	2.28 ± 0.40
*H2-Q2*	1.53 ± 0.13 ^a^	2.75 ± 0.43 ^b^	*Psmb8*	1.4 ± 0.09 ^c^	1.92 ± 0.28 ^b^
*H2-Q4*	1.33 ± 0.17 ^c^	1.94 ± 0.34 ^b^	*Psmb9*	1.28 ± 0.15 ^c^	1.58 ± 0.14 ^b^
*H2-Q7*	1.49 ± 0.20 ^c^	2.67 ± 0.30 ^b^	*Psmb10*	1.59 ± 0.37 ^a^	2.12 ± 0.31 ^b^
*H2-Q10*	1.39 ± 0.21 ^c^	2.44 ± 0.18 ^b^	*Psme2*	1.36 ± 0.22 ^c^	1.62 ± 0.35 ^b^

^a^ altered expression level, wild type *versus rsh* or *msd* (fold change ≥ 1.5 and *FDR* < 0.1); ^b^ altered expression level, *rsh versus msd* (*FDR* < 0.1); ^c^ comparable expression level, wild type *versus rsh* (fold change < 1.5 or *FDR* > 0.1).

In addition to induction of genes in the MHC class I and II regions on chromosome 17, we find a number of interferon-related immune genes located on other chromosomes that induced in *rsh* and *msd* mice, mostly with greater induction in *msd* (Table 4). These include the *Irf1* and *Irf8* transcriptional activators of interferon-responsive genes and interferon-responsive genes β_2_-microglobulin (*β2m*), which interacts with MHC proteins, *Ifit1*, *Ifit3*, *Ifitm3*, *Ifitm7*, *Isg15*, the low and high affinity IgG Fc receptor genes (*Fcgr1–3*) and the macrophage scavenging receptor (*Fcrls*), which suggests the activation of microglia and peripheral immune cells. Further, the induction of chemokine families *Ccl2*, *5*, *19* and *Cxcl1*, *10* likely reflect signaling to microglia and *Lcn2* induction suggests M1 polarization of these cells in *msd* mice [45].

Induction of *Ccl19* is important for trafficking of immune cells into the parenchyma and T-cell homing to secondary lymphoid organs and *Ccl5* induction promotes macrophage infiltration and is known to exacerbate immune-mediated demyelination [46]. *Cxcl1* attracts neutrophils into the parenchyma and *Cxcl10* recruits monocytes, natural killer cells and T-cells by inducing expression of cell adhesion molecules such as ICAM-1 on endothelial cells, which is induced in *msd* mice. Induction of *Cd52* in *rsh* and *msd* mice, which is a lymphocyte marker and target of Alemtuzumab under FDA consideration for the treatment of MS [47], indicates extravasation of these cells into the CNS and induction of *Cd68* is consistent with the CD68 immunofluorescence staining on activated microglia in Figure 2. Finally, induction of the *Csf1r*, *Csf2r*, *Trem2*, *Dap12/Tyrobp*, *Ship*/*Inpp5d* and *Stat1* genes are associated with intracellular signaling in myeloid cells such as dendritic cells and macrophages.

From the perspective of innate and adaptive immune system involvement in disease associated with supernormal expression of *PLP1*, several recent studies show that the CD8^+^ T cell proteins, perforin 1 and granzyme B, as well as the microglia/macrophage protein, sialoadhesin, exacerbate the phenotype of *Plp1*-overexpressor mice [48,49]. Expression of the genes encoding either of these T cell proteins is not detected on the *rsh* or *msd* microarrays, and expression levels of *Sialoadhesin* are comparable to wild type controls. Thus, at this stage we can exclude these molecules as playing a significant role in the pathology of the missense *Plp1* mutants.

**Table 4:** Expression fold changes of other non-chromosome 17 interferon-induced genes in microarray data for *rsh* and *msd* mice.

Gene	Fold Change		Gene	Fold Change	
	*rsh*	*msd* ^a^		*rsh*	*msd* ^a^
*Irf1*	1.3 ± 0.26 ^c^	1.79 ± 0.14 ^b^	Ccl19	1.13 ± 0.17 ^c^	1.77 ± 0.23 ^b^
*Irf8*	1.64 ± 0.32 ^a^	1.87 ± 0.20 ^b^	Cxcl1	1.25 ± 0.17 ^c^	1.86 ± 0.10 ^b^
* 2m*	1.95 ± 0.50 ^a^	2.77 ± 0.54 ^b^	Cxcl10	3.95 ±1.51 ^a^	8.66 ± 2.92 ^b^
*Ifit1*	1.52 ± 0.31 ^a^	2.47 ± 0.46 ^b^	Lcn2	1.2 ± 0.04	3.01 ± 0.25 ^b^
*Ifit3*	1.86 ± 0.41 ^a^	2.84 ± 0.49 ^b^	Icam1	1.31 ± 0.24 ^c^	1.68 ± 0.17 ^b^
*Ifitm3*	1.3 ± 0.14	1.5 ± 0.20	Cd52	2.25 ± 0.39 ^a^	2.59 ± 0.84
*Ifitm7*	1.28 ± 0.16	1.86 ± 0.45 ^b^	Cd68	1.79 ± 0.26 ^a^	1.98 ± 0.28
*Isg15*	1.38 ± 0.23	1.92 ± 0.13 ^b^	Csf1r	1.4 ± 0.21 ^c^	1.66 ± 0.18 ^b^
*Fcgr1*	2.0 ± 0.26 ^a^	2.43 ± 0.11 ^b^	Csf2r	1.47 ± 0.35 ^c^	1.56 ± 0.41
*Fcgr2*	1.3 ± 0.27 ^c^	1.68 ± 0.32	Trem2	3.43 ± 0.62 ^a^	3.39 ± 0.92
*Fcgr3*	1.59 ± 0.49 ^a^	1.84 ± 0.33 ^b^	Tyrobp	2.44 ± 0.69 ^a^	2.24 ± 0.5
*Fcrls*	2.03 ± 0.39 ^a^	2.38 ± 0.48	Inpp5d	1.4 ± 0.22 ^c^	1.51 ± 0.23
*Ccl2*	1.52 ± 0.51 ^c^	2.69 ± 0.39 ^b^	Stat1	1.38 ± 0.25 ^c^	1.90 ± 0.33 ^b^
* cl5*	1.23 ± 0.17 ^c^	1.71 ± 0.21 ^b^			

^a^ altered expression level, wild type *versus rsh* or *msd* (fold change ≥ 1.5 and *FDR* < 0.1); ^b^ altered expression level, *rsh versus msd* (*FDR* < 0.1); ^c^ comparable expression level, wild type *versus rsh* (fold change < 1.5 or *FDR* > 0.1).

### 2.6. Steroid and Isoprenoid Pathways Are Downregulated in *rsh* and *msd* Mice

Although expression levels of more than 60% of the dysregulated genes in *rsh* and *msd* mice are at least 1.5-fold lower than controls, few KEGG pathways are enriched in a DAVID analysis using this gene subset. The steroid and isoprenoid backbone biosynthesis pathways associated with cholesterol synthesis are exceptions, and we observe broad reductions in most of the constituent genes including the two rate limiting enzymes, *Hmgcr* (*rsh*, 0.77 ± 0.13; *msd*, 0.57 ± 0.06) and *Hmgcs* (*rsh*, 0.75 ± 0.05; *msd*, 0.64 ± 0.03). The results likely signify a lack of induction of these important pathways, which are necessary during the first few postnatal weeks of normal development to myelinate retinal ganglion axons in the optic nerve. Thus, differentiating oligodendrocytes in *rsh* and *msd* mice data fail to upregulate cholesterol synthesis for myelinogenesis because of the negative impact of severe metabolic stress and the UPR.

Because *rsh* mice synthesize approximately 50% of the normal amount of myelin [50] and *msd* mice synthesize about 5% [41] during development, we expect that these pathways should be more strongly reduced in *msd*. Indeed, the lack of *Hmgcr* induction in *rsh* (*i.e.*, <1.5-fold change) is consistent with a significant amount of myelin membrane synthesized by these mutants. Nevertheless and consistent with hypomyelination in the mutants, we observe lower expression levels of the major myelin genes (Table 2): *Plp1*, *Mbp*, *Mog*, *Cnp1*, *Cldn11* and *Mag*.

### 2.7. Mitochondrial and Oxidative Stress Response Pathways Are Not Broadly Transcriptionally Dysregulated in *msd* and *rsh* Mice

The flip side of Figure 5 showing 644 genes dysregulated in *rsh* and *msd* mice, is that 7117 genes (>90%) are expressed at relatively normal levels (fold change < 1.5). A DAVID analysis of these genes reveals a number of enriched pathways (*FDR* < 0.1), but from the perspective of protein misfolding and metabolic stress, which characterizes disease in *rsh* and *msd* mice and is often coupled with oxidative stress for many diseases [51], the absence of transcriptional changes in almost all of the nuclear-encoded mitochondrial genes and the major antioxidant/oxidative stress genes represented on the microarrays is unexpected. Table 5 and the text below summarizes relevant data from the microarrays and suggests that oxidative stress is not a significant contributing factor to disease in *rsh* and *msd* mice, at least at P16.

The major transcription factors regulating nuclear-encoded mitochondrial proteins are PGC-1, NRF1/2, TFB1/2 and Tfam [52]. Nrf2 (encoded by *Nfe2l2*), TFB2 (*Tfb2m*) and Tfam are detectable on the microarrays and expressed at normal levels in *rsh* and *msd* mice, although Taqman PCR shows that *Nfe2l2* is induced by 1.5-fold in these mutants (*p* < 0.05). Despite this induction, mitochondrial target gene expression profiles (nuclear-encoded) are normal in the microarrays, including: all of the NADH dehydrogenase subunits *Ndufa*, *b*, *c*, *s*, *v* (36/38 subunits) in Complex I; the succinate dehydrogenase subunits *a*–*d* (4/4) in Complex II; all of the cytochrome c reductase subunits (10/10) in Complex III; cytochrome c itself; the 10 nuclear cytochrome c oxidase subunits *Cox4*–*8* in Complex IV and accessary subunits Cox*11*–*20* (15/17); and *Atpase* type *F* (15/19) and all type *V* subunits (13/13) in Complex V. In addition, mitochondrial RNA polymerase (*Polrmt*) and the voltage-dependent anion channels, *Vdac1–3*, are expressed at normal levels.

In addition to the prominent role of Nrf2 in the transcriptional regulation of mitochondrial biogenesis [52], this protein is also important in regulating the oxidative stress response by dimerization with Maf family coactivators and binding to antioxidant response elements (*ARE*) in many genes [53]. Some Nrf2 *ARE* targets are induced in *rsh* and/or *msd* mice including sestrin 2 (*Sesn2*), peroxiredoxin (*Prdx6*) and glutathione peroxidase (*Gpx*); however, expression levels of well-known and widely characterized oxidative stress pathway proteins, NF-κB, Cu/Zn-superoxide dismutase (*Sod1/2*) and catalase (*Cat*), are not altered in either mutant. Thus, we find little evidence for a robust or broad-based oxidative stress response in the mutant mice although induction of several genes in the pathway suggests an initiation of stress signaling, at least in *msd* mice.

### 2.8. Is Immune Activation in PMD Likely to Precede or Follow Mutant PLP1 Expression and UPR Induction in Oligodendrocytes?

In spite of a thorough understanding of the etiology of PMD, it is difficult to define with certainty, a detailed timeline at the tissue and cellular levels that can account for all aspects of the pathophysiology, including whether-or-not immune system activation is upstream or downstream of UPR induction. Nevertheless, we can employ the tenets of causality and Occam’s razor to construct plausible and testable postulates. Our working model for PMD in the current study is that coding region *PLP1* mutations induce the UPR and perturb oligodendrocyte homeostasis [19,20], which comprises the cellular pathology detected by the innate immune system. Although we know from the work of Popko and colleagues [33] that cytokines can induce the UPR in oligodendrocytes, it is unclear why immune cells in PMD patients would secrete cytokines prior to detecting pathology. In this regard, Figure 3 shows that microglia/macrophages in gray matter regions from *rsh* and *msd* mice are in a resting state, presumably in the absence of pathology. Thus, because altered homeostasis in white matter stems from UPR activation, causality would dictate that microglia/macrophage activation is secondary to UPR induction. Data from other studies also show that UPR induction in many cell types triggers downstream immune activation [25] and indeed, even in infectious disease, immune activation is consequential, not a precedent, to pathology.

**Table 5:** Expression fold changes of antioxidant and oxidative stress pathway genes in microarray data for *rsh* and *msd* mice.

Gene	Expression Fold-Change Compared to Controls				
	Microarrays		Taqman PCR		
	*rsh*	*msd*		*rsh*	*msd*
*Nfe2l2*	1.03 ± 0.19	1.15 ± 0.13		1.53 ± 0.12 ^b^	1.5 ± 0.08 ^b^
*Tfb2m*	1.02 ± 0.12	0.99 ± 0.15		*n.m.*	*n.m.*
*Tfam*	1.13 ± 0.03	1.14 ± 0.07		*n.m.*	*n.m.*
*MafF*	1.11 ± 0.15	1.21 ± 0.16		*n.m.*	*n.m.*
*Sesn2*	2.85 ± 0.42 ^a^	2.56 ± 0.51 ^a^		*n.m.*	*n.m.*
*Prdx6*	1.1 ± 0.25	1.50 ± 0.28 ^a^		*n.m.*	*n.m.*
*Gpx*	1.24 ± 0.24	1.57 ± 0.40 ^a^		*n.m.*	*n.m.*
*RelA*	1.06 ± 0.14	1.03 ± 0.10		1.61 ± 0.05 ^b^	1.04 ± 0.10
*Sod1*	1.06 ± 0.23	1.15 ± 0.24		*n.m.*	*n.m.*
*Sod2*	1.02 ± 0.19	1.01 ± 0.16		*n.m.*	*n.m.*
*Cat*	0.81 ± 0.17	0.91 ± 0.12		*n.m.*	*n.m.*

^a^ fold change ≥ 1.5 and *FDR* < 0.1; ^b^ fold change ≥ 1.5 and *p* < 0.05; *n.m.*, not measured.

### 2.9. A Genetically Defined Model to Examine Secondary Activation of the Immune System in Disease

The use of whole genome mRNA-based microarray analyses to identify global transcriptional changes that could shed light on the molecular mechanisms of neurodegenerative diseases have been a major focus for more than a decade. Many studies involve patient tissue or animal models where the primary lesion is believed to arise in neurons, including Alzheimer, Parkinson, Huntington diseases, amyotrophic lateral sclerosis (ALS), schizophrenia and stroke. Several studies have also examined tissue from MS patients or EAE. The overwhelming conclusion from these studies has been the prominence of immune system activation and oxidative stress responses stemming from induced Nrf2 transcription factor expression in the pathophysiology of these diseases [51,54,55,56,57,58]. From the perspective of the current study, one of the major disadvantages of many studies is a lack of knowledge about the primary insult so that it is unclear if immune involvement and oxidative stress constitute primary or secondary events in the pathogenesis.

In the current study, we have taken a genomics approach to examine the effects of early stage pathology on gene regulation in a genetically-defined mouse model of PMD using a structurally simple tissue that is strongly affected by disease stemming from the accumulation of misfolded *Plp1* gene products in oligodendrocytes. We chose the age of the mice at P16 to coincide with steady-state middle-stage disease where hypomyelination and cell death are prominent features of pathology but renewal from proliferative oligodendrocyte precursors is sufficient to maintain normal numbers of differentiating cells [20,59]. Our data reveal broad based induction of interferon and TNF related genes, activation of microglia and possible extravasation of peripheral immune cells into the CNS in *msd* mice. However, we find more limited induction of immune pathways in *rsh*. Accordingly, these data provide an important conceptual framework for considering the pathophysiology associated with missense mutant protein accumulation and activation of the UPR in oligodendrocytes leading to secondary degenerative processes in the CNS.

The UPR is a highly conserved signaling pathway in eukaryotes that retains functional similarities from yeast to primates, albeit with greater complexity in multicellular organisms to protect organisms against novel insults as they occupy ever-more varied environmental niches. While early studies in mammalian cells have focused on UPR activation by viral glycoproteins, fungal toxins, ionophores and genetic defects, more recently identified induction mechanisms include dietary- or autoimmune-mediated insults [33,60,61,62]. 

With regard to the current study, the intriguing possibility that proinflammatory cytokine release in the CNS can induce the UPR in oligodendrocytes and potentially underlie the pathogenesis of diseases such as MS [13] has prompted us to consider the reverse process; where UPR induction in oligodendrocytes could activate the innate immune system, leading to proinflammatory cytokine release and the generation of a positive feedback loop, increasing oligodendrocyte damage and eventually autoimmune pathology. Indeed, we have previously reported that the UPR is active in oligodendrocytes from *rsh* and *msd* mice as well as in normal appearing white matter from an MS patient [19].

This notion is not without support from the literature. One study has demonstrated UPR induction in demyelinating lesions in MS [63] and two recent case reports show that PMD involving missense mutations in the *PLP1* gene can be clinically indistinguishable from MS and can mimic autoimmune pathology in the sense that clinical symptoms are ameliorated by steroid treatment [64,65]. Further, a study in transgenic rats overexpressing PLP1 in oligodendrocytes shows evidence of CD8^+^ T cell restriction in spinal cord [66], suggesting secondary inflammation and autoimmune activation following a primary insult in oligodendrocytes.

Although not exhaustive, the microarray data reported herein demonstrate that broad changes in immune-associated signaling pathways occur in response to a nonimmune etiology, with distinct disease severity-dependent profiles that paint a somewhat rudimentary landscape of innate and adaptive immune responses to oligodendrocyte dysfunction and/or death. Because microglia are arguably the first responders to many types of brain injury [34], it is likely that these cells initiate signals leading to cytokine/chemokine secretion in the CNS by direct release of some of these molecules and/or by releasing non-peptide metabolites such as ATP or UTP to communicate with, and activate astrocytes, endothelial cells and other cell types [67].

Although we find evidence for cytokine/chemokine signals that can recruit monocytes to the CNS in the *Plp1* mutants, we have not determined the proportion of CD68^+^ cells that are derived from infiltrating cells. A previous study has examined this question in the allelic *Plp1* mutant *jimpy* mouse [68], which exhibits severe disease roughly comparable to *msd* mice [69]. Vela and colleagues [68] show that the majority of microglia/macrophages likely arise from the proliferation of endogenous cells rather than from peripheral blood. By extrapolation to the current study, we speculate that most CD68^+^ cells in *msd* mice arise from endogenous cell populations. In contrast, other studies have observed significant numbers of peripheral immune cells in *Plp1* mutants, with the most detailed analysis performed on *Plp1* overexpressor mice, but these animals were 5–12 months of age [32]. Tatar and colleagues [22] also observed an age-dependent increase in immune activation, which may account for the relatively mild level of immune activation in *msd* mice, which were P16. Unfortunately, the lifespan of these mutants is 3–4 weeks, which may be insufficient time for robust immune responses such as CNS invasion by peripherally activated T and/or B cells.

### 2.10. Is UPR Pathology Associated with Other Autoimmune Responses in Different Tissues?

In the current study, we demonstrate that a primary trafficking defect of mutant *PLP1* gene products in oligodendrocytes of PMD patients and mouse models can secondarily activate an innate immune response leading to at least partial activation of the adaptive immune system. It remains to be determined whether altering the genetic background of *rsh* and *msd* mice favors further immune activation and can trigger inflammatory disease. In this light, our findings prompt a broader question. Can protein trafficking defects in other cell types or tissues also provoke autoimmune responses leading to inflammatory or degenerative processes?

For several diseases, there is considerable affirmative evidence [70]. Ankylosing spondylitis is a common multi-system inflammatory disease for which a major risk factor is the wild type HLA-B27 haplotype. HLA-B27 is expressed by macrophages and normally traverses the secretory pathway to the cell surface as a trimeric complex, with β_2_-microglobulin and bound peptide, to present the peptide to immune cells. However, even under normal physiologic conditions, a significant proportion of HLA-B27 nascent chains fail to adopt a stable higher-ordered structure and induce the UPR. Through an as yet unclear mechanism, HLA-B27 misfolding leads to elevated IL-23 production, which triggers T cells to adopt a T_h_17 phenotype and leads to inflammation and autoimmunity. Interestingly, polymorphisms in the IL-23 receptor constitute a second risk factor for ankylosing spondylitis and may predispose for the disease in HLA-B27 individuals by altered signaling in T cells upon ligand binding. Thus, the primary insult is metabolic stress, which secondarily induces cytokine production and drives T cells toward autoimmune activity in multiple organs.

Another example of autoimmunity as a secondary disease has been shown in C57Bl/6 mice. In this model, the pathophysiology associated with inducible, transgene-mediated expression of the major histocompatibility complex (MHC) class I protein, H-2K^b^, in skeletal muscle involves activation of the UPR as H-2K^b^ accumulates in the ER [71]. Thereafter, the mice develop inflammatory myositis, indicating that the immune response is secondary to induction of metabolic stress in the muscle cells. Importantly, the early accumulation of MHC I protein in the muscle cells of patients with idiopathic inflammatory myopathies parallels the pathology observed in the transgenic mice and provides a more satisfying account of pathogenesis than does the consideration of inflammation as the primary insult [72].

### 2.11. Uncoupling of Metabolic Stress from Oxidative Stress in *rsh* and *msd* Mice

An unexpected finding to emerge from the microarray analyses is the relative absence of transcriptional changes to nuclear-encoded mitochondrial genes and antioxidant gene signaling pathways in *rsh* and *msd* mice, which suggests that oxidative stress is not initially a significant factor in the pathophysiology. This finding contrasts with the conclusions emerging from studies of well-known protein misfolding and metabolic stress neurodegenerative diseases such as Alzheimer, Huntington and Parkinson diseases and ALS, that oxidative stress drives disease pathology [51]. Currently, we do not have a clear explanation to account for our observations; however, the purpose of this study design was to analyze UPR signaling in a steady-state middle phase of disease rather than in advanced end-stage disease as is often the case for patient studies. Accordingly, we may have separated the processes of metabolic stress and oxidative stress and we suggest that the latter pathology may be secondary to protein accumulation in the endoplasmic reticulum. Looking to the future of therapeutics design for neurodegenerative diseases, perhaps efforts should be focused on mitigating disease by means other than alleviating oxidative stress (*i.e*., upstream processes). In this regard, a number of recent clinical trials designed to reduce oxidative stress in the major neurodegenerative diseases generally show poor efficacy in patients despite promising pre-clinical results [57,58].

## 3. Experimental Section

### 3.1. Animals

Male mice from two naturally-occurring mutant strains, *rsh* and *msd*, and their wild type littermates maintained on a B6C3F1/Tac genetic background are examined in this study. A third strain, *Plp1#66*, that was maintained on a C57BL6/J genetic background was included as a mouse model of *PLP1* duplication in PMD patients [22]. The *rsh* mutation is an isoleucine-to-threonine change at amino acid 187 (I187T) in the *PLP1* gene [73] and causes moderate hypomyelination with pronounced tremors, ataxia, rarely seizures and a normal life span. The *msd* mutation is an alanine-to-valine change at codon 243 (A243V) [74] and causes severe hypomyelination with pronounced tremors, ataxia, seizures and a life span of 21–30 days. These animals are excellent models of PMD and the specific mutations have direct correlates in humans [75,76]. We also used hemizygous and homozygous *Plp1#66* overexpressor mice [77] at P25 as previously characterized positive controls for cytokine and chemokine expression profiles in response to primary oligodendrocyte pathology [22].

The *rsh* and *msd* female carrier mice have been backcrossed to B6C3F1 male mice (Taconic Farms, Hudson, NY, USA) for at least 15 generations. As previously reported, these mutants develop initial symptoms by 11–13 days postnatally (P11–13) and all experiments have been performed at P16. The *Plp1#66* mice are maintained on a C57BL6/J (Jackson Laboratories, Bar Harbor, MA, USA) background and were used at P25 following the manifestation of behavioral symptoms in response to CNS pathology [22]. All experiments reported here have been approved by the Wayne State University IACUC committee.

### 3.2. PMD Autopsy Specimens

Tissue slices (10 × 3 mm cross-sections) comprised of gray and white matter around the U-fibers of human temporal lobe were dissected during autopsy (9–24 h postmortem intervals) and immersion fixed in either 10% formalin or freshly prepared 4% paraformaldehyde in 0.1 M phosphate buffer pH 7.2 for several weeks at 4 °C before processing into paraffin blocks or overnight infiltration with 25% sucrose in PBS for cryostat sectioning. All tissue samples were obtained under an HIC approved protocol.

### 3.3. Immunofluorescence Labeling

Mice were anesthetized with avertin (375 mg/kg i.p.) and perfused using an intracardiac catheter under gravity flow for 15 min with freshly-prepared, room temperature, 4% paraformaldehyde in 0.1 M sodium phosphate buffer, pH 7.2. In general, mouse cryostat sections from paraformaldehyde-perfused mice were permeabilized with methanol at −20 °C for 10 min and blocked for 30 min in 2% normal goat serum, 1% BSA, 0.1% gelatin in TBS [78]. An exception was made for anti-CD68 labeling, where sections were not permeabilized because methanol masks the epitope. Sections were immunolabeled with the following antibodies overnight at room temperature: rabbit anti-Iba-1 (1:200, WAKO Chemicals USA, Richmond, VA, USA) and rat anti-CD68 (1:200, Abd Serotec, Raleigh, NC, USA). Sections were washed with 1× PBS and incubated with appropriate Alexa-conjugated secondary antibodies (Invitrogen, Carlsbad, CA, USA) for 3 h, washed twice in PBS, labeled with DAPI and mounted. Then, 10 μm sections of human brain autopsy specimens embedded in paraffin were dewaxed, hydrated, treated with 1× TUF fluid (Zymed, Carlsbad, CA, USA) as recommended for antigen retrieval and immunolabeled using the same procedure as above. Antibodies used were: mouse anti-MBP (1:1000, SMI99, Sternberger Monoclonal Inc., Baltimore, MD, USA); rabbit anti-Iba-1; monoclonal anti-human CD68 antibody (clone 514H12, AbD Serotec, Oxford, UK).

### 3.4. Immunofluorescence Image Processing

Immunolabeled sections mounted on glass slides were photographed using an ORCA-R^2^ camera (Hamamatsu Corporation, Bridgewater, NJ, USA) on a Leica DMRA2 microscope (Leica Microsystems, Bannockburn, IL, USA) configured for epifluorescence or VT*_i_* spinning-disk confocal (Visitech International, Newtown, PA) microscopy with HeNe and Argon lasers. Raw, 16 bit gray scale images for each fluorophore (DAPI, Alexa488, 568 and 647) were combined in Photoshop CS4 (Adobe Systems, San Jose, CA, USA). We used two types of masks for fluorescence images, autofluorescence masks and DAPI masks.

The red channel was used to capture autofluorescence in human autopsy specimens and mask the intensity of this artifact in the green channel during image processing. To do this, the gray scale autofluorescence image was pasted into the adjacent layer above the RGB micrograph. The lookup table was inverted (*i.e.*, white = 0, black = 65,536) then “Multiplied” with the RGB layer. The mask opacity was generally 100% but could be used to refine the strength of the autofluorescence masking. The gray scale DAPI image was pasted into a layer above the RGB micrograph to “Lighten” the nuclei with 25%–30% opacity. This effect adds a modicum of white color to improve print visibility.

### 3.5. Cell Counts and Statistics

To determine the proportion of Iba-1-labeled microglia/macrophages that were CD68^+^ activated cells, we counted cells in white matter regions of transverse mouse spinal cord sections. All major white matter tracts exhibited similar activation of these cells; however, spinal cord offers clear advantages for quantification of activated cells shown in Table 1. One 40× field from each of dorsal, ventral, left and right lateral funiculi were counted in four or five sections per mouse, with three wild type, *rsh* or *msd* mice analyzed per genotype. A single *Plp1*#66 mouse was analyzed. Approximately 400–500 Iba-1^+^ cells per animal were counted. The average proportions of Iba-1/CD68 double-positive microglia/macrophages from each anatomical region were similar in each section and the data were pooled to yield an overall average per mouse. Data for each mouse per genotype were then averaged (mean ± S.D.) for statistical analysis using ANOVA with Bonferroni post hoc testing (Graphpad Software Inc., La Jolla, CA, USA).

### 3.6. Mouse Tissue Dissections, Microarrays and Taqman PCR Analyses

Mice aged P13, 16 or 21 were decapitated and optic nerve was rapidly dissected, rinsed in PBS pH 7.4 and frozen in round bottom polypropylene tubes (Thermo Fisher Scientific Inc., Waltham, MA, USA). For microarray analysis, three pools of optic nerves from 10 to 20 mice per genotype at P16 were collected and total RNA was purified through CsCl as previously described [79]. Four genotypes were included: male *rsh* mice and male littermate controls and male *msd* mice with male littermate controls. Samples were subject to quality control using electrophoresis on a 2100 Bioanalyzer Model G2938B (Agilent Technologies, Santa Clara, CA, USA), labeled with Cy5-CTP (PerkinElmer, Waltham, MA, USA) and hybridized in triplicate to whole mouse genome G4122A oligonucleotide arrays (12 arrays in total) according to the manufacturer recommendations using a pooled-reference standard labeled with Cy3-CTP (PerkinElmer) to account for sample and technical variability.

For quantitative Taqman PCR analysis of gene expression, liquid nitrogen flash-frozen optic nerve samples from individual mice stored at −80 °C were homogenized in 1 mL of QIAzol Lysis Reagent (Qiagen Inc., Valencia, CA, USA) at full speed for 1 min using an ultra tarrax T25 with a micro probe (IKA Works Inc, Wilmington, NC, USA). Total RNA was then purified using an RNeasy Lipid tissue mini kit (Qiagen). Each sample was then subjected to quality control, including: concentration determination at 260 nm, spectroscopic absorbance ratioing at 260/280 nm and 28 *S*/18 *S* ratioing. Finally, two-point standard curves were generated to ensure sample linearity from 0.4 μg to 2 μg aliquots of total RNA in reverse transcriptase reactions (Life Technologies Corp., Carlsbad, CA, USA) using TATA-binding protein (*Tbp*) expression (see below for assay details).

Purified spinal cord RNA samples were stored frozen at −80 °C and cDNAs were reverse transcribed using 2 μg aliquots. For gene expression analysis, cDNAs were thawed, diluted 10- or 25-fold and in water for 25 μL Taqman assays in triplicate in a 384 well format. The Taqman probes used in this project are: *Tbp*, Mm00446973_m1; *Plp1*, Mm01297210_m1; *Mbp*, Mm01262037_m1; *Mog*, Mm00447824_m1; *Chop*, Mm00492097_m1; *Atf3*, Mm00476032_m1; *Atf4*, Mm00515324_m1; *Noxa*, Mm00451763_m1; *Trib3*, Mm00454880_m1; *4ebp1*, Mm01962435_g1; *Bip*, Mm00517691_m1; *Nfe2l2*, Mm00477784_m1.

### 3.7. Quantification of Microarray Expression Data

Laserscanner (Agilent Technologies) images of the microarray chips were quantified to generate gene expression data using ImaGene 6.0. software (Biodiscovery Inc., Hawthorne, CA, USA). Probe spots were assigned flag = 2 if the signal-to-noise ratio < 2 (*i.e*., probe signal/local background < 2) and 3 if contamination was found using automated default parameters in the software. The probe spots were also manually inspected to identify probe spot contamination. The signal medians of the ImaGene data were imported into GeneSpring software (Agilent Technologies) and transformed using LOWESS normalization. Examples of these data transformations are shown in Supplemental Figure 1. Expression data and subsequent ontology analyses were subjected to t-tests and ANOVA, assuming unequal variance, and *P* values for significance were corrected using a Benjamini false discovery rate (*FDR*) = 0.1.

### 3.8. Bioinformatics Analyses of Microarray Expression Data

All 60mer DNA probe sequences for the Agilent 4122A microarray were downloaded from the Agilent website and mapped to the mouse genome (mm9) to obtain an updated list of the represented genes. Expression data from all genes represented on the microarrays were assembled into an Excel spreadsheet and sorted into Changed Gene subgroups according to fold changes over controls ≥ 1.5, FDR < 0.1. This fold-change was arbitrarily chosen, but is viewed as meaningful using several categories: biological relevance; technical relevance for microarrays; and technical relevance for verification by Taqman PCR and northern blotting.

Gene names and accession IDs from the Changed Gene subgroups were parsed using Gene List Manager portal in the DAVID Bioinformatics Database v6.7 [42] and enrichment analysis was used to identify KEGG metabolic and signaling pathways of potential interest. The *M. musculus* transcriptome within DAVID was used as the reference (Background) gene set for the enrichment analysis. Statistically significant gene enrichment in KEGG pathways was assessed using Benjamini *FDR* < 0.1.

To identify chromosomal expression hotspots within which changes to gene expression were predominately coordinately regulated by either induction or suppression, we generated a background histogram of all expressed probes as a function of chromosomal location and overlaid histograms of six major subgroups of differentially expressed genes: common to *rsh* and *msd* changed up or down (*r* ∩ *m ↑*; *r* ∩ *m ↓*), unique to *rsh* changed up or down (*r* ⊄ *m ↑*; *r* ⊄ *m ↓*) and unique to *msd* changed up or down (*m* ⊄ *r ↑*; *m* ⊄ *r ↓*).

## 4. Conclusions

In the current study, we have demonstrated that neurodegenerative disease associated with primary activation of the UPR can lead to secondary activation of the innate and adaptive immune system and the possible recruitment of peripheral immune cells to the CNS. Our study joins a growing body of evidence in the literature indicating that autoimmune diseases of poorly characterized or unknown etiology could be initiated by metabolic stress as the primary insult. In addition, we show that steady state disease arising from metabolic stress can be uncoupled from antioxidant and oxidative stress pathways, which has important implications for the large number of published studies showing that reactive oxygen species and oxygen radical production drives disease.
